# Effects of Acidic Polysaccharides from *Gastrodia* Rhizome on Systolic Blood Pressure and Serum Lipid Concentrations in Spontaneously Hypertensive Rats Fed a High-Fat Diet

**DOI:** 10.3390/ijms13010698

**Published:** 2012-01-11

**Authors:** Ok-Hwan Lee, Kyung-Im Kim, Chan-Kyu Han, Young-Chan Kim, Hee-Do Hong

**Affiliations:** 1 Department of Food Science and Biotechnology, Kangwon National University, Chuncheon 200-701, Korea; E-Mail: loh99@kangwon.ac.kr; 2 Department of Hotel Culinary Arts and Food Service, Hyejeon College, Hongseong 350-702, Korea; E-Mail: kikim69@hanmail.net; 3 Korea Food Research Institute, Seongnam, Kyonggi 463-746, Korea; E-Mails: ckhan@kfri.re.kr (C.-K.H.); yckim@kfri.re.kr (Y.-C.K.)

**Keywords:** *Gastrodia* rhizome, acidic polysaccharide, serum lipid level, blood pressure

## Abstract

The effects of acidic polysaccharides purified from *Gastrodia* rhizome on blood pressure and serum lipid levels in spontaneously hypertensive rats (SHR) fed a high-fat diet were investigated. Acidic polysaccharides were purified from crude polysaccharides by DEAE-Sepharose CL-6B. Thirty-six male SHR were randomly divided into three groups: *Gastrodia* rhizome crude polysaccharide (A), acidic polysaccharide (B) groups, and a control group (C). A 5-week oral administration of all treatment groups was performed daily in 3- to 8-week-old SHRs with a dose of 6 mg/kg of body weight/day. After 5 weeks of treatment, total cholesterol in the acidic polysaccharide group, at 69.7 ± 10.6 mg/dL, was lower than in the crude polysaccharide group (75.0 ± 6.0 mg/dL) and the control group (89.2 ± 7.4 mg/dL). In addition, triglyceride and low-density lipoprotein cholesterol levels in the acidic polysaccharide group were lower than in the crude polysaccharide and control groups. The atherogenic index of the acidic polysaccharide group was 46.3% lower than in the control group. Initial blood pressure after the initial three weeks on the high-fat diet averaged 195.9 ± 3.3 mmHg among all rats. Compared with the initial blood pressure, the final blood pressure in the control group was increased by 22.8 mmHg, whereas it decreased in the acidic polysaccharide group by 14.9 mmHg. These results indicate that acidic polysaccharides from *Gastrodia* rhizome reduce hypertension and improve serum lipid levels.

## 1. Introduction

Hypertension, also known as high blood pressure, is a major public health problem due to its high prevalence and complications [1]. It is defined as a systolic blood pressure of 140 mmHg or higher, and a diastolic blood pressure of 90 mmHg or higher [2]. One of the factors contributing to the development of hypertension is life style and eating habits, such as high consumption of salt, alcohol, cholesterol and fat [3]. Over consumption of salt, alcohol, cholesterol and fat have resulted in alarming increases in the incidences of hypertension, insulin resistance, and obesity [4]. On the other hand, current research suggests that fruits and vegetables contain polysaccharides and phytochemicals that substantially lower blood pressure and blood lipid levels [5,6]. In addition, several reports have been published on anti-hypertensive effect of natural polysaccharide and presented the following: the polysaccharides fraction from American ginseng berry extract has a significant anti-hyperglycemic activity [7]. Treatment with polysaccharide fraction of mushroom species such as *Pleurotus nebrodensis* in SHR showed antihypertensive effect via the inhibition of angiotensin conversion [8]. We previously reported [9] that the anti-hypertensive action of macromolecules such as crude polysaccharides from *Gastrodia* rhizome in SHR is significantly stronger than that of the protein fraction and low-molecular-weight fraction (phenolic compound).

*Gastrodia* rhizome, a polysaccharide-enriched dry tuber of *Gastrodia elata* Blume (Orchidaceae), has been used in traditional medicine in Korea, Japan and China as an anti-convulsant, an analgesic, and a sedative against general paralysis, epilepsy, vertigo, and tetanus [10]. The major physiological substances of *Gastrodia elata* Blume are gastrodin, parishin, and vanillyl alcohol, glycoprotein, in addition to polysaccharides, including α-d-glucan [11–13]. Among the components of *Gastrodia elata* Blume, glycoproteins inhibit platelet aggregation and exhibit antithrombosis activity [11]. Tong and others [14] reported that sulfated α-d-glucan from *Gastrodia elata* Blume exerts a potent inhibitory effect on dengue virus serotype 2. Although *Gastrodia* rhizome has various biological actions [15–18], the antihypertensive effect of acidic polysaccharides purified from *Gastrodia* rhizome in SHR fed a high-fat diet has not yet been studied.

This study therefore investigated the antihypertensive potential of acidic polysaccharides purified from *Gastrodia* rhizome and also evaluated the concentrations of serum lipids, including total cholesterol (TC), triglyceride (TG), high-density lipoprotein (HDL) cholesterol, and low-density lipoprotein (LDL) cholesterol in SHR fed a high-fat diet.

## 2. Results and Discussion

### 2.1. Characterization of Acidic Polysaccharides from Gastrodia Rhizome

**Figure 1:**
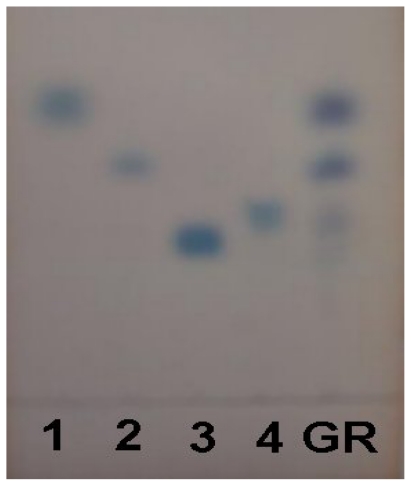
Thin-layer chromatography (TLC) patterns of acid hydrolyzate of acidic polysaccharides from *Gastrodia* rhizome. 1: xylose; 2: glucose; 3: galacturonic acid; 4: glucuronic acid; GR: acid hydrolyzate of acidic polysaccharides from *Gastrodia* rhizome.

In our previous study [9], a hot-water extract of *Gastrodia* rhizome was fractionated to obtain a crude polysaccharide (GR-0). The crude polysaccharide exhibited the most potent antihypertensive action compared with the protein fraction and low-molecular-weight fraction [9]. It was further fractionated by ion-exchange chromatography on DEAE-Sepharose CL-6B. As shown in Table 1, crude polysaccharide (GR-0) contained 1.19 ± 0.11% of total protein, 86.25 ± 1.54% of total sugar, and 12.56 ± 1.15% of acidic polysaccharides, whereas the acidic polysaccharide fractions of DEAE-Sepharose CL-6B contained 0.32 ± 0.02% of total protein, 82.40 ± 1.16% of total sugar, and 17.28 ± 0.58% of acidic polysaccharides, respectively. The fraction of acidic polysaccharides from *Gastrodia* rhizome might consist of simple monosaccharide of six carbon atoms. According to the retention factor, the fraction of acidic polysaccharides included xylose, glucose, galacturonic acid, and glucuronic acid (Figure 1).

### 2.2. Antihypertensive Effect of Acidic Polysaccharides from Gastrodia Rhizome

**Figure 2:**
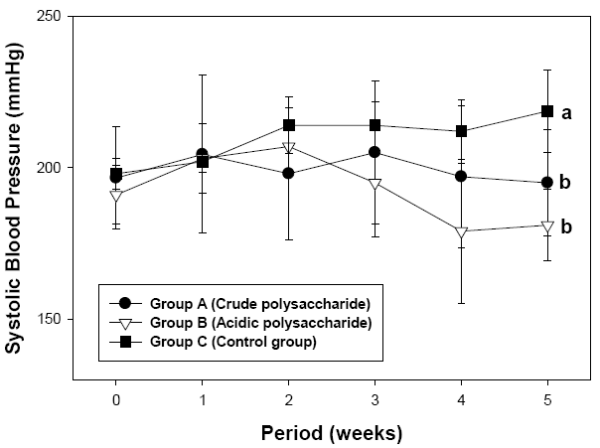
Effects of crude and acidic polysaccharides of *Gastrodia* rhizome on blood pressure after 5 weeks of treatment. * *p* < 0.05 indicates statistically significant different to only control group (Group C); also data are presented as the mean ± standard deviation of twelve experiments in each group.

**Figure 3:**
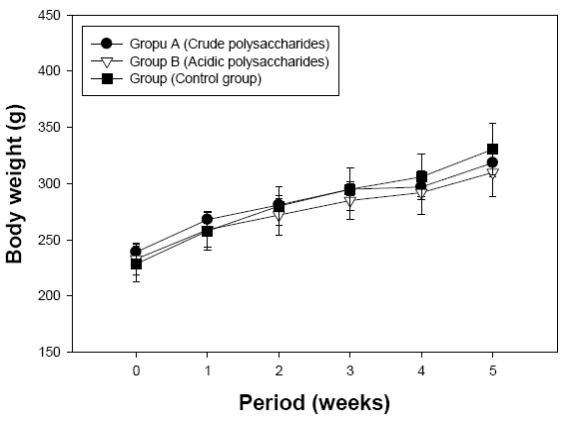
Effects of crude and acidic polysaccharides of *Gastrodia* rhizome on body weight after 5 weeks of treatment. No significant difference was observed between Groups A and B and control (Group C) at *p* > 0.05. The body weight was recorded weekly during the 5 weeks experimental period. Values are expressed as the mean ± standarddeviation.

To determine the effect of acidic polysaccharides on hypertension in SHR fed a high-fat diet, systolic blood pressure was evaluated. Reference blood pressure (RBP), after consuming the high-fat diet for an initial 3 weeks, averaged 195.9 ± 3.3 mmHg. Figure 2 shows that the blood pressure of Group C (control) increased from 195.9 ± 3.3 mmHg to 218.7 ± 13.6 mmHg in 5 week of treatment period. However, the blood pressure of Group A and Group B was 195.0 ± 17.6 and 181.0 ± 11.87 mmHg, respectively, in the same period. The crude and acidic polysaccharide groups showed significantly lower blood pressure than the control group (*p* < 0.05). Compared with the initial blood pressure (195.9 mm Hg), the final blood pressure of the control group increased by 22.8 mmHg, but that of the acidic polysaccharide group decreased by 14.9 mmHg. As shown in Figure 3, no significant difference (*p* > 0.05) was observed in body weight between both polysaccharide groups and the control group during the 5-week treatment period. Our results are consistent with those of Zhu and others [19], who reported that the d-polymannuronic sulfate markedly reduces systolic blood pressure (SBP) and diastolic blood pressure (DBP) dose-dependently and decreases heart rate (HR) with a reduction in arterial blood pressure. Ding and others [11] showed that polysaccharide 2-1 faction from *Gastrodia elata* has remarkable anticoagulation and anti-thrombosis effects in mice and *in vitro* models. The antihypertensive activity of Group A and B may depend on the amount of acidic polysaccharides in the fractions (Table 1 and Figure 2).

### 2.3. Effects of Acidic Polysaccharides from Gastrodia Rhizome on Serum Lipid Levels

As shown in Table 2, serum TC was higher in the control group, at 89.2 mg/dL, than in both *Gastrodia* rhizome groups (group A, 75.0 mg/dL and group B, 69.7 mg/dL). Similarly, the administration of crude and acidic polysaccharides significantly decreased TG by 20.2 and 21.4% and LDL-cholesterol by 14.7 and 22.4%, respectively, compared to the control group. However, HDL-cholesterol in SHR treated with crude and acidic polysaccharides significantly increased by 6.7 and 14.8%, respectively. At the same time, AI in the acidic polysaccharide group was 46.3% lower than in the control group.

Our results reveal that both crude (Group A) and acidic polysaccharides (Group B) are able to reduce blood pressure (Figure 2), TC, TG, and LDL (Table 2) in SHR fed a high-fat diet. After 5 weeks of treatment, blood pressure (181.0 ± 11.8 mmHg) in SHR treated with acidic polysaccharides was lower than in the crude polysaccharide (195.0 ± 17.6 mmHg) and control groups (218.7 ± 13.6 mmHg). Compared with the control group, the administration of the acidic polysaccharides significantly decreased blood pressure by 17.2% (Figure 2). Furthermore, Group B decreased TC by 21.9%, TG by 21.4%, LDL-cholesterol by 22.4%, and AI by 46.3% (Table 2). In addition, serum HDL-cholesterol was markedly higher in the Group B, at 31.0 ± 2.0 mg/dL, than in the control (Group C, *p* < 0.05). During the experimental period, no significant differences were observed in body weight (Figure 3), food intake, or water intake (data not shown) between either polysaccharide groups or the control group.

Hypertension is one of the risk factors for stroke, which is associated with age, gender, elevated cholesterol, smoking, alcohol, excessive weight, and family history [20]. Recently, antihypertensive agents were suggested by various mechanisms. Inhibition of angiotensin I converting enzyme (ACE), one of antihypertensive mechanism, has been used for evaluating antihypertensive activity of natural products and functional food [21]. ACE removes a dipeptide from the C terminus of angiotensin I to form angiotensin II, a highly reactive hypertensive compound [20]. A few reports have investigated that polysaccharide from food ingredients present hypertensive activity via inhibition of ACE activity. Miyazawa and others [8] showed that polysaccharide fraction of mushroom species such as *Pleurotus nebrodensis* in SHR reduced blood pressure via the inhibition of angiotensin conversion. In addition, pectin hydroxamic acid shows the dose-dependent inhibitory activity of ACE [20]. Therefore, our results also suggest that the administration of the acidic polysaccharide distinctly retarded the development of hypertension in SHR via inhibition of ACE activity. However, these enzyme assays need to be examined in the future to better understand the mechanism of acidic polysaccharides from *Gastrodia* rhizome on antihypertensive activity.

## 3. Experimental Section

### 3.1. Materials

The *Gastrodia* rhizome was purchased from the Herbal Medicine Co-operative of Muju, Chonbuk, South Korea. Cleaned *Gastrodia* rhizome was peeled, thinly sliced, and dried at 40 ± 5 °C for 6 h. The dried *Gastrodia* rhizome was ground to 20–30 mesh using a grinder (IKA M 20, IKA, Staufen, Germany).

### 3.2. Preparation of Crude and Acidic Polysaccharides

**Figure 4:**
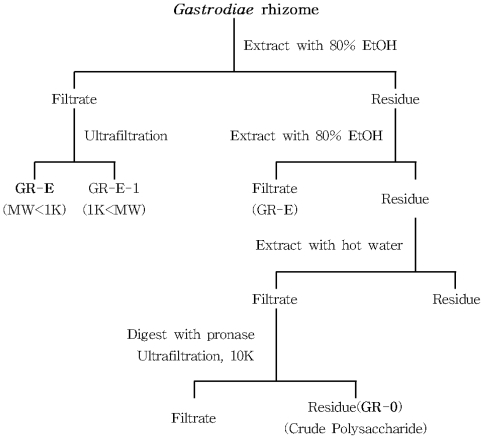
Isolation and preparation of crude polysaccharides from *Gastrodia* rhizome. Content of figure is adapted from Hong and others [9,17].

**Figure 5:**
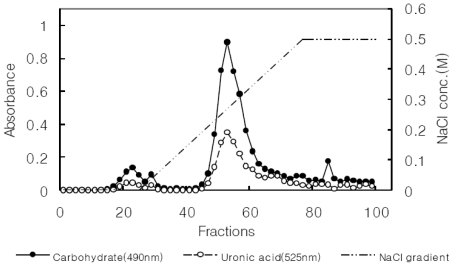
Ion-exchange chromatography patterns on DEAE-Sepharose CL 6B of acidic polysaccharides from *Gastrodia* rhizome.

The *Gastrodia* rhizome powder was refluxed with 10 volumes (v/w) of 80% ethanol at 60 °C for 3 h, and the extraction was repeated two times. The extracts were filtered through Whatman filter paper (No. 2). The precipitates were extracted with 2 L boiling distilled H_2_O for 3 h. The aqueous extract was centrifuged at 2000× *g* for 15 min and filtered through Whatman filter paper (No. 2). Hot water extract was dissolved in 1 L of Tris-HCl buffer (pH 7.5) that contained 10 mM CaCl_2_, and the extract was treated with 50 mg of pronase. The reaction mixture was incubated at 37 °C for 6 days. The reaction was stopped by boiling for 5 min, followed by dialysis. The nondialyzable portion, crude polysaccharides (GR-0), was lyophilized to obtain the pronase-digested product (Figure 4). The crude polysaccharide extract was further purified using ion-exchange chromatography on a DEAE-Sepharose CL-6B (Figure 5). The crude polysaccharides were eluted by stepwise elution with 0, 0.05, 0.1, 0.2, 0.3, 0.4, and 0.5 M NaCl. It was dissolved in drinking water and used for animal test samples. Total sugar and protein contents of crude and acidic polysaccharides were determined using phenol-H_2_SO_4_ [22] and the BCA method with a BCA™ protein assay kit with BSA as the standard (Thermo Scientific, Pittsburgh, PA, USA).

### 3.3. Determination of Acidic Polysaccharides Profiles

The profiles of acidic polysaccharides from *Gastrodia* rhizome were determined by a modified method of Thetsrimuang and others [23]. The acidic polysaccharides (3–4 mg) were hydrolyzed with 2 N HCl at 100 °C for 2–6 h. The hydrolyzed sample and standard monosaccharide were analyzed on the same thin-layer chromatography (TLC) aluminum sheet pre-coated with silica (Silica gel 60 F254, Merck, Germany). The developing agent was a mixture of acetonitrile and water (85:15, v/v). The monosaccharides were visualized on the plate after dipping into 0.5% α-naphthol in 5% H_2_SO_4_ and heating until they appeared as a dark spots. Xylose, glucose, galacturonic acid and glucuronic acid were used as standard monosaccharide.

### 3.4. Animal Experiments and Diet

Thirty-six male SHR (4 weeks old) were purchased from Charles River Laboratories, Inc. (Yokohama, Japan) and used after 1 week of quarantine and acclimation. Animals were kept in the animal facility of the Korea Food Research Institute in a light-controlled room (12-h light/dark cycle) at an average temperature of 24 °C and humidity of 60%. This experiment was conducted in facilities approved by the Guiding Principles for the Care and Use of Laboratory Animals of the Ethics Committee of the Korea Food Research Institute. All rats were fed an AIN-93M-based diet supplemented with high fat (10% lard, 2% corn oil, and 1% cholesterol) as the source of increased fat (Table 3). All rats were provided *ad libitum* access to the high-fat diet (HFD) and tap water throughout the 8-week treatment period. After three weeks on the HFD, the rats were randomly divided into three groups: *Gastrodia* rhizome crude polysaccharide (A) and acidic polysaccharide (B) groups and control group (C) fed only the high-fat diet. As shown in Table 4, both crude polysaccharide (6 mg/kg) and acidic polysaccharide (6 mg/kg) were administered by oral gavage to SHR. The dose of crude and acidic polysaccharides were set by considering effective dose of antihypertensive fractions of hot water and crude polysaccharide ranging from 3.6 to 14.7 mg/kg of body weight/day in SHRs [9,24]. Acidic polysaccharides were consisting of 12.56% in fraction of crude polysaccharide. Samples were administered by oral gavage in 1 mL/250 g of body weight on a daily basis 7 days per week for 5 weeks. The control animals were treated with the same volume of distilled water.

### 3.5. Blood Pressure Measurements

Blood pressure was measured by a modified method of [25] in a radio frequency-shielded room. After the stabilization of animals in a 29 ± 1 °C box for 10 min, the tail systolic blood pressure was measured with a blood pressure monitor (Muromachi Kikai MK-2000, Tokyo, Japan) as the mean value of three consecutive measurements.

### 3.6. Blood Chemistry

At the end of the treatment, all rats fasted for 12 h were anesthetized with isoflurane and euthanized, and arteriovenous blood was collected. Blood samples were examined for serum lipid levels and atherogenic index. The concentrations of TC, TG, HDL cholesterol, and LDL cholesterol were determined using a commercial kit (Asan Phamaceutical Co., Seoul, Korea). Atherogenic index (AI) was calculated from the formula: (TC-HDL)/HDL.

### 3.7. Statistical Analysis

Data are presented as the means ± standard deviation. The results were statistically analyzed by one-way ANOVA and Student’s *t*-tests. Statistical significance was accepted at a level of *p* < 0.05.

## 4. Conclusions

The tuber of *Gastrodia elata* Blume, *Gastrodia* rhizome, has been traditionally used as a folk medicine to treat headache, hypertension, migraine, dizziness, epilepsy, infantile convulsion and tetanus in Korea and China [12,16,26]. It is also certified for use in food by the Korean Food and Drug Administration [27]. We previously reported [9] that macromolecules such as polysaccharides from *Gastrodia* rhizome significantly reduce systolic blood pressure and improve serum lipid profile in SHR fed a high-fat diet. However, the efficacy of purified polysaccharides, such as acidic polysaccharides, from *Gastrodia* rhizome as an antihypertensive agent has not been investigated. Therefore, we further purified the acidic polysaccharides from *Gastrodia* rhizome by ion-exchange chromatography on DEAE-Sepharose CL-6B, and evaluated the effects of acidic polysaccharides on anti-hypertensive effect and serum lipid levels in SHR fed a high-fat diet. Purified acidic polysaccharides consisted mainly of xylose, glucose, galacturonic acid, and glucuronic acid. The acidic polysaccharide fraction from *Gastrodia* rhizome markedly reduced systolic blood pressure in SHR fed a high-fat diet. Furthermore, the acidic polysaccharides positively regulated serum lipid levels in SHR. These results suggest that the acidic polysaccharides from *Gastrodia* rhizome might be excellent natural antihypertensive and anti-atherosclerosis agents due to their biological properties. These new findings help fill a critical gap between epidemiological observations and clinical studies on the antihypertensive benefits of acidic polysaccharides from *Gastrodia* rhizome. Further mechanism studies are needed to address this important issue.

## Figures and Tables

**Table 1:** Chemical compositions and yields of the crude polysaccharides and the acidic polysaccharide fractions purified from *Gastrodia* rhizome.

	Yield (g)	Total protein (%)	Total sugar (%)	Acidic polysaccharides (%)
Crude polysaccharides fraction	2.47	1.19 ± 0.11	86.25 ± 1.54	12.56 ± 1.15
Acidic polysaccharide fraction	0.61	0.32 ± 0.02	82.40 ± 1.16	17.28 ± 0.58

**Table 2:** Effects of crude and acidic polysaccharides from *Gastrodia* rhizome on serum lipid levels and AI in spontaneously hypertensive rats (SHR) fed a high-fat diet.

Group (*n* = 12)	Lipids (mg/dL) and AI				

	TC 1	TG 2	HDL 3	LDL 4	AI 5
A	75.0 6 ± 6.0 ^b^	133.0 ± 9.4 ^b^	28.8 ± 1.6 ^b^	24.4 ± 1.8 ^b^	1.61 ± 0.31 ^b^^7^
B	69.7 ± 10.6 ^b^	131.0 ± 8.7 ^b^	31.0 ± 2.0 ^a^	22.2 ± 3.6 ^b^	1.24 ± 0.22 ^c^
C	89.2 ± 7.4 ^a^	166.7 ± 16.3 ^a^	27.0 ± 0.9 ^b^	28.6 ± 3.8 ^a^	2.31 ± 0.27 ^a^

1 TC: total cholesterol;

2 TG: triglyceride;

3 HDL: high-density lipoprotein cholesterol;

4 LDL: low-density lipoprotein cholesterol;

5 AI: atherogenic index ((TC-HDL)/HDL);

6 Values are mean ± SD (*n* = 12);

7 Means in the same column with different superscript letters are significantly different (*p* < 0.05).

**Table 3:** Composition of the experimental diet based on the AIN-93 diet with high fat.

Ingredients	Content (%)
Casein (feed grade CP 85%)	20.00
Corn starch	39.75
Dextrinized corn starch	13.20
Sucrose	10.00
Soybean oil	7.00
Cellulose (fiber)	5.00
Mineral mixture 1	3.50
Vitamin mixture 2	1.00
l-Cystine	0.30
Choline bitartrate	0.25

1 Contained per kg mixture: CaHPO_4_ 500 g, NaCl 74 g, K_3_C_6_O_7_·H_2_O 220 g, K_2_SO_4_ 52 g, MgO 24 g, 48% Mn 3.5 g, 17% Fe 6.0 g, 70% Zn 1.6 g, 53% Cu 0.3 g, KIO_3_ 0.01 g, CrK(SO_4_)_2_·12H_2_O 0.55 g and sucrose.

2 Contained per kg mixture: thiamin·HCl 600 mg, riboflavin 600 mg, pyridoxine·HCl 700 mg, nicotinic acid 3 g, vitamin A 400,000 IU (retinyl acetate), vitamin E (dl-α-tocopheryl acetate) 5000 IU, vitamin D_3_ 2.5 mg, vitamin K 5.0 mg and sucrose [9,17].

**Table 4:** Experimental design.

Group (*n* = 12)	1st phase (3 weeks)	2nd phase (5 weeks)
**A**^1^	HFD 2	Crude polysaccharides + HFD
**B**	HFD	Acidic polysaccharides + HFD
**C**	HFD	HFD

1 Groups A and B were orally administered crude and acidic polysaccharides, respectively, of *Gastrodia* rhizome at a concentration of 6 mg/kg, and Group C was orally administered with the same volume of distilled water, using a stainless-steel oral tube for 5 weeks.

2 HFD (high-fat diet): AIN diet-based commercial rat chow containing 10% lard, 2% corn oil, and 1% cholesterol (w/w).
